# Sternalis Muscle: A Cadaveric Case Report of a Rare Variant With Multiple Branching

**DOI:** 10.7759/cureus.68263

**Published:** 2024-08-31

**Authors:** Mohammed Bahgat, Abdul Sattar Khan, Amira E Alsemeh

**Affiliations:** 1 Anatomy Division, Biomedical Sciences Department, College of Medicine, King Faisal University, Al-Ahsa, SAU; 2 Family and Community Medicine Department, College of Medicine, King Faisal University, Al-Ahsa, SAU; 3 Human Anatomy and Embryology Department, Faculty of Medicine, Zagazig University, Zagazig, EGY

**Keywords:** cadaveric case report, anatomy, muscle variations, chest wall variations, rectus sternalis, sternalis

## Abstract

The sternalis muscle is a rare anatomical variant located in the anterior thoracic wall. Understanding variations in the sternalis muscle anatomy is essential for clinicians, especially radiologists and surgeons to prevent misdiagnosis and avoid complications during surgical procedures in the anterior thoracic region. We present a unique case of bilateral branched sternalis muscles. On either side, the sternalis muscle lies deep to the breast and superficial fascia and superficial to pectoralis major muscle and pectoral fascia. Each sternalis muscle is branched into medial and lateral slips with the medial slip larger than the lateral slip. The medial slip of the right sternalis was larger than the medial slip of the left sternalis. The lateral slip of the left sternalis was larger than the lateral slip of the right sternalis. The lateral slip of the left sternalis muscle has a curved course with superior-lateral convexity and inferomedial concavity. Near its middle, the left sternalis lateral slip is branched into two smaller slips separated by a narrow cleft. The left sternalis muscle in our report is a new variant with multiple branching, which cannot be matched to any type of the previously described classifications. In this case report, we discussed the need of modification of the currently available sternalis classification system to accommodate all types of the previously reported sternalis muscles including the branching pattern of this muscle.

## Introduction

Many muscle variations are found during dissection of human cadavers. Sternalis muscle is one of these variations in the pectoral region lateral to the sternum, deep to superficial fascia, and superficial to pectoralis major muscle [1]. As mentioned by Turner (1867), this muscle was first observed by Struthers in 1604 and first described in detail by DU Puy in 1726 [2]. Although, this muscle has been termed with 21 synonyms [1], the most commonly used name is the sternalis muscle [2,3,4,5,6] followed by rectus sternalis [7,8,9].

There is great variation in description of all features of this muscle including variable origin, insertion, nerve supply, dimensions, and variable forms [4,5,6]. The variation also included classification of different variants of the sternalis muscle by different authors [1,5,10,11,12]. The prevalence of sternalis showed great variations in different countries ranging from 1.0% to 1.3 % in Taiwanese up to 23.7% in Chinese [1,5,10,13]. Despite its rarity, many authors have concerns related to the clinical significance of the sternalis muscle as it may mimic pathologic conditions such as breast masses or lymphadenopathy on imaging studies or lead to problem during surgery in the pectoral region [7,9,10,11,13].

The prevalence of the sternalis in the living patient using radiographs depends on the methods of the study. Although it is very low in mammographic studies (ranging from 0% to 0.02%) [10], studies in MDCT imaging showed incidence nearly similar to cadaveric studies [13,14]. Here, we present a case of a sternalis muscle with a new pattern of branching, which has not been previously reported in the literature.

## Case presentation

A 55-year-old female cadaver revealed two anomalous muscles (right and left) in the pectoral region lateral to the sternum, consistent with sternalis muscle. Away from these two sternalis muscles, the anterior thoracic wall appeared normal with no other anatomic variant or abnormality detected in the pectoral muscles. The morphology and topography of the two sternalis muscles were documented, with photographs captured with a digital camera and measurements performed with the aid of a digital sliding caliper. 

**Figure 1:**
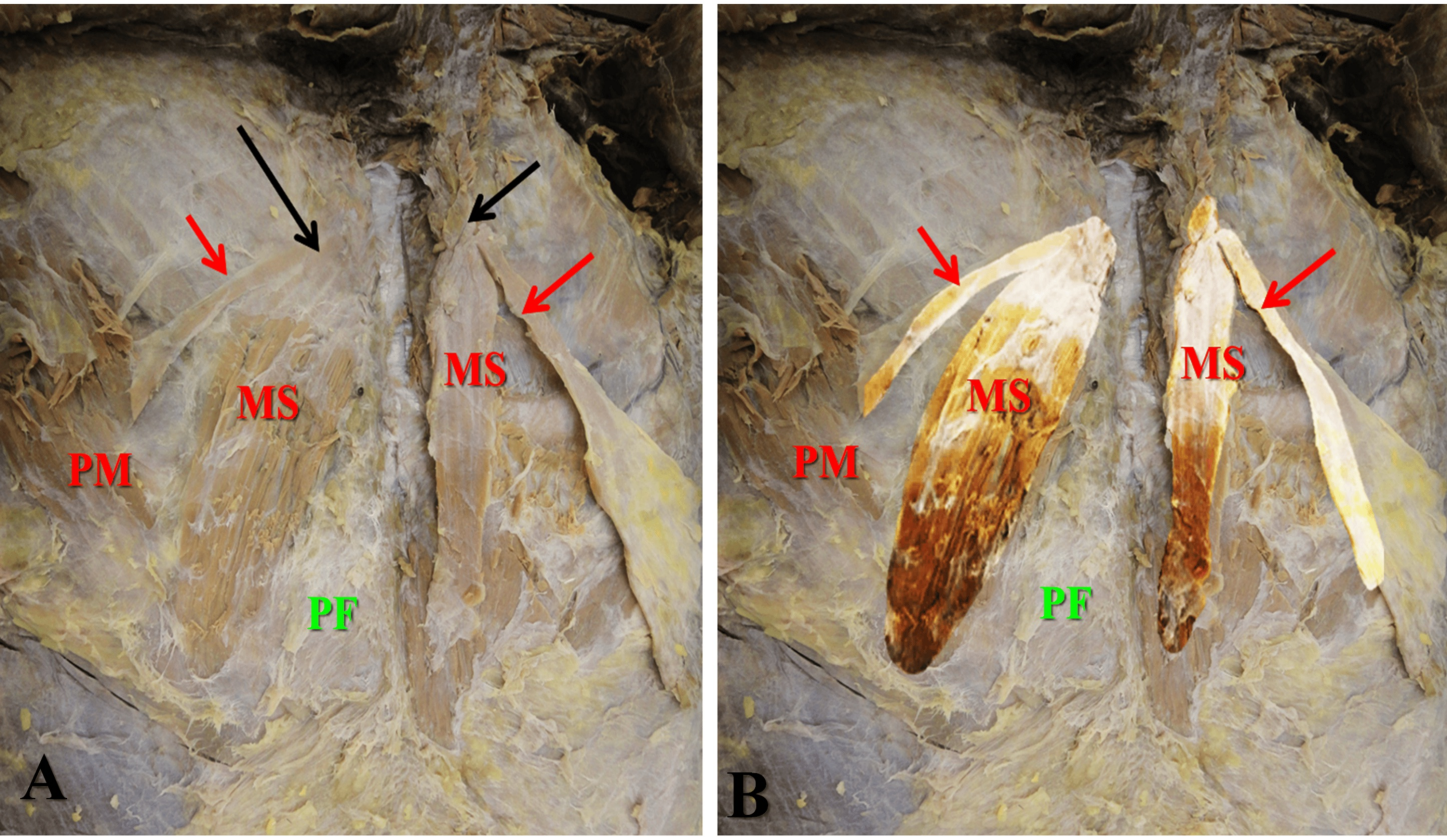
(A) After removal of the breasts and superficial fascia, there is bilateral branched sternalis muscle (black arrows). (B) Same photo with highlighting of the muscles. Each muscle has two slips, medial slip (MS) and lateral slip (red arrows). The medial slip is larger than the lateral slip on both sides. Comparing similar slips of the two muscles, the medial slip is larger on the right muscle, while the lateral slip is larger on the left muscle PM: Pectoralis major muscle; PF: pectoral fascia

Each sternalis muscle lies deep to the breast and superficial fascia and superficial to pectoralis major muscle and pectoral fascia (Figure 1). On the right side, the medial slip was broader than the lateral slip, descended downward and laterally adjacent to the sternum making 30º angle with the midline. The length of the medial slip was 13 cm, and its breadth was 3.4 cm. The lateral slip was much shorter and thinner and descended downward and laterally making 60º angle with the midline and 30º angle with the medial slip. The length of the lateral slip was 6.3 cm, and its breadth was 0.6 cm.

**Figure 2:**
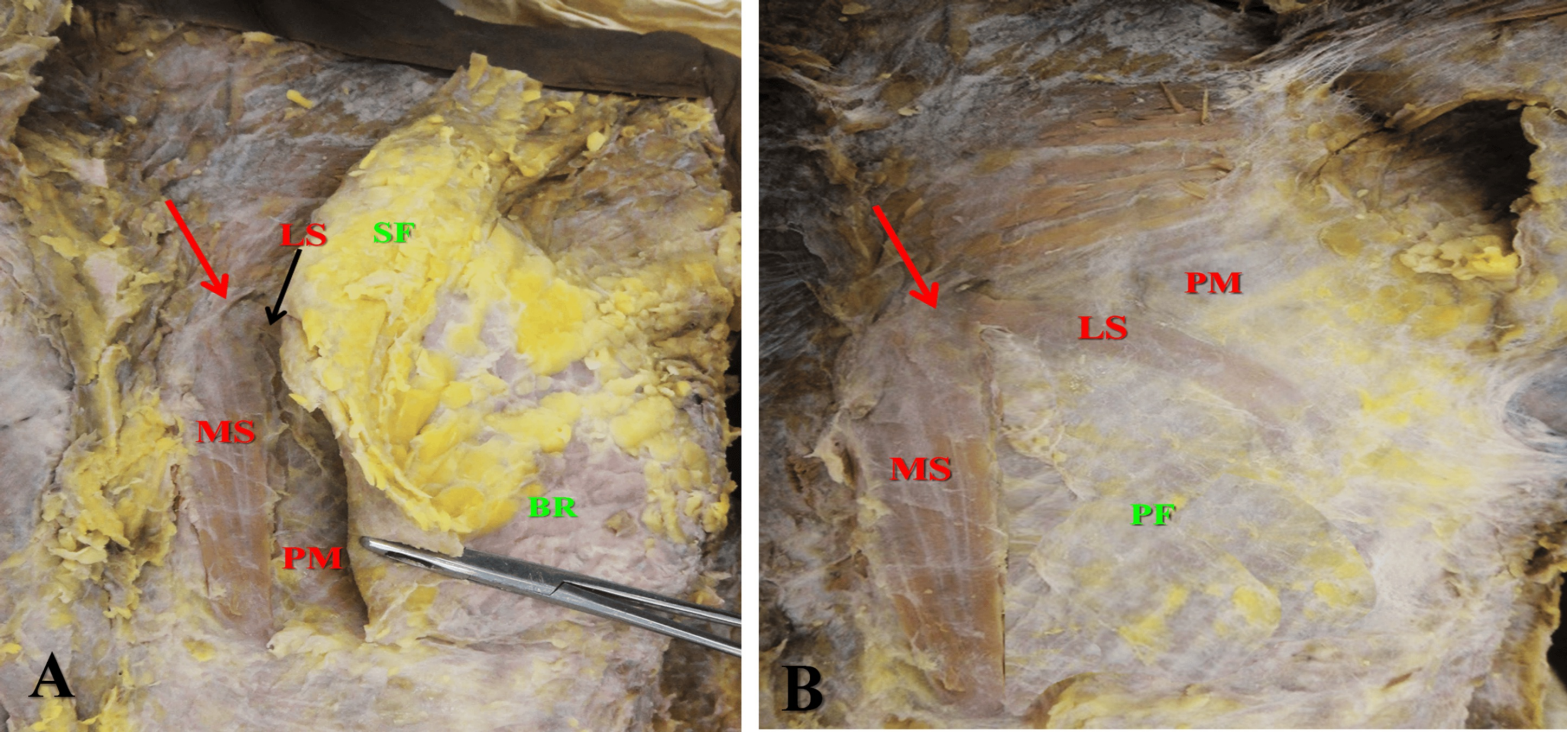
Left sternalis muscle. (A) Partial removal of the superficial fascia and breast. (B) Complete removal of the superficial fascia and breast. The left sternalis muscle (red arrow) lies deep to the superficial fascia (SF) and breast (BR) and superficial to the pectoralis major muscle (PM) and pectoral fascia (PF). The sternalis muscle consists of two slips; a straight broader and longer medial slip (MS) and a curved thinner and shorter lateral slip (LS). Red arrow marks the upper end of the muscle

On the left side, the medial slip was also broader than the lateral slip, which descended vertically adjacent and parallel to the sternum (Figures 1, 2). The length of the medial slip was 10.8 cm, and its breadth varied in its upper, middle, and lower parts (2.2, 2.0, and 1.0 cm, respectively). The lateral slip was much thinner and has a curved course with superior-lateral convexity and inferomedial concavity (Figure 2B). It extended from the upper part of the vertical band downward and to the left (Figure 2B) making 65º angle with the medial slip.

**Figure 3:**
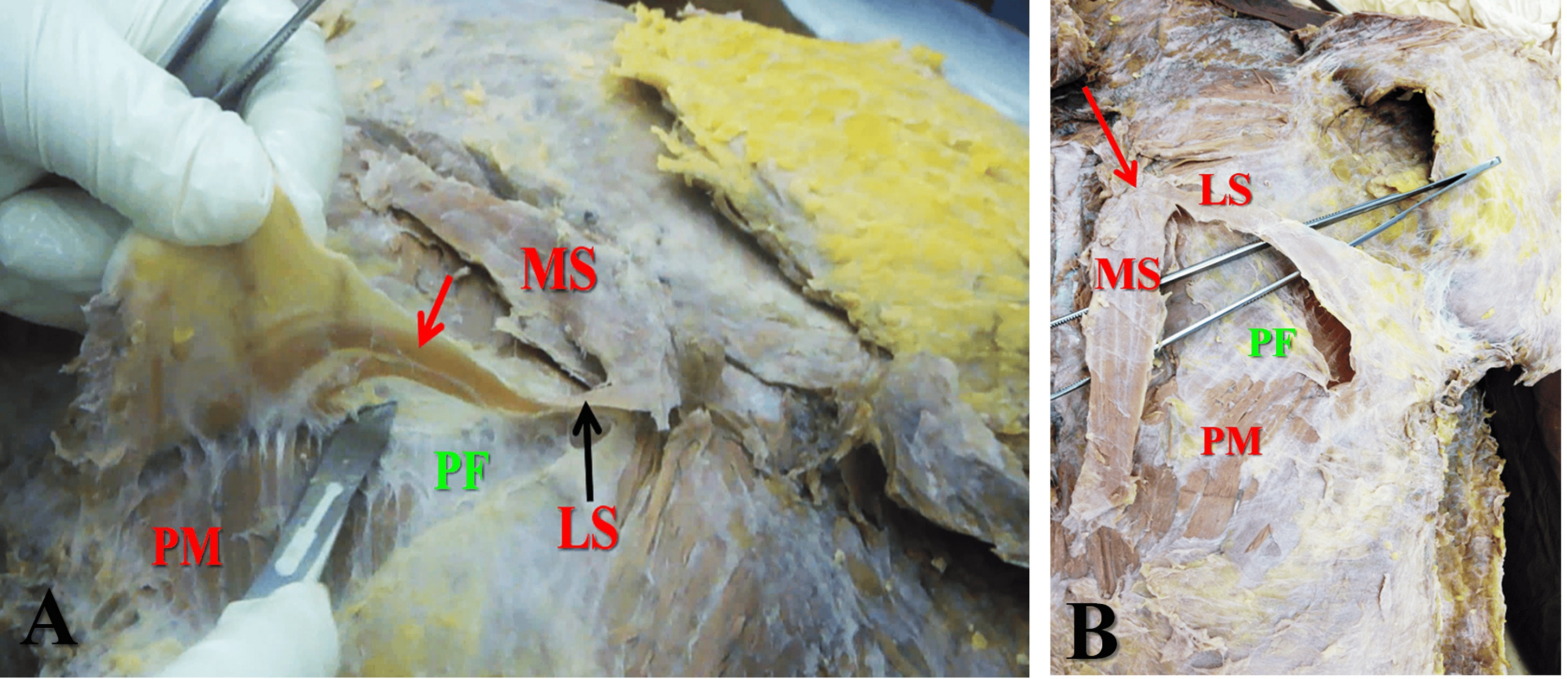
(A) The lateral slip (LS) is dissected from the pectoral fascia (PF). The lateral slip is branched into two smaller branches near its middle (red arrow). (B) The medial slip (MS) and lateral slip of the left sternalis muscle are separated from the pectoral fascia (PF) and pectoralis major muscle (PM). Red arrow marks the upper end of the muscle

Unlike previously described cases, the left sternalis muscle exhibited a new pattern of branching. The branching started few mm below its upper end as widely separated and unequal medial and lateral slips. There is another branching of the lateral slip, which started near its middle as two smaller slips separated by a narrow cleft (Figures 2B, 3A). The whole length of the lateral slip was 9.3 cm, and its breadth was 0.9 cm in its upper part and 1.1 cm just before its branching.

## Discussion

We presented a case of bilateral branched sternalis muscles characterized by the beginning of the branching a few millimeters below its upper end. Moreover, the lateral slip of the left muscle starts rebranching about its middle. Based on many published case reports, research articles, and reviews, there are different attempts to classify the previously reported sternalis muscles according to their different forms, bilateral or unilateral, single or double, present on one or both sides, crossing the midline or not [1,5,10,11,12]. The sternalis muscle, presented in our report, cannot be completely matched to any type of these classifications. Type II 3 in Jelev et al.'s (2001) classification [5] is the most one resembling our case with some differences. First, the lateral slip of Jelev type II 3 passes horizontally with an upward concavity, while the lateral slip of the left sternalis in our case descends downward and to the left with upward and lateral convexity. The second difference is the rebranching of the left sternalis lateral slip which cannot be found in any of the previous classifications.

Although there is an extensive classification of the sternalis muscles, new forms still emerge that do not fit with any of the previous classification types. The new sternalis variant of our report is an example and is supported by Dudgeon et al. (2017) who reported bilateral sternalis with a new variant on the right side. They reported a mixed type-right triple sternalis muscle, with single bicipital converging and single bicipital diverging sternalis muscle [15]. In another case report, Prall et al. (2019) presented a bilateral case with the left sternalis muscle having a triceps converging the upper end and biceps diverging the lower end [16]. These reports and our report support the need for a modified or new classification which can accommodate all previously described sternalis muscles.

Moreover, nothing mentioned about the incidence of the different forms/types in previously described classifications. However, it seems that the simple type with a single muscle adjacent and parallel to the sternum is the most frequent type [1-9,11,12]. This is considered as lacking important information which is required to evaluate the impact of the presence of the sternalis muscles on clinical practice especially the interpretation of mammograms.

The size of our case's right sternalis was larger than the left one, especially the medial slip. A larger sternalis muscle on the right side is also reported by many authors [6,11]. Recently Li et al. (2024) reported a bilateral sternalis (simple form) with a larger right sternalis muscle and a very small left sternalis muscle [17].

Many authors have concerns related to the clinical significance of sternalis muscle as it may mimic pathologic conditions such as breast masses or lymphadenopathy on mammographic studies or lead to problem during surgery in the pectoral region [1,7,9,10,11,13,18]. However, there are some features which help in the accurate identification of the sternalis muscle in mammography and prevent misdiagnosis as breast lesion. First, the sternalis appears only in the craniocaudal mammographs and cannot be seen in mediolateral mammographs. Second is the position of the sternalis behind the breast in the posteromedial part of the craniocaudal mammograph. Third, the awareness of the radiologist with sternalis muscles helps them to include this muscle in the differential diagnosis of a mass seen in the posteromedial part of the craniocaudal mammographs [10]. As multidetector computed tomography (MDCT) imaging studies in the living patient showed incidence nearly similar to cadaveric studies [13,14], and hence better identification of sternalis muscle, MDCT can be used to differentiate between this muscle and breast lesion if confusion persists.

The sternalis vertical part (medial slip) of our report is similar to the simple type of sternalis previously described in many literatures [1-9,11-13]. It appears as a band of muscle just lateral to the sternum on either or both sides. The sternalis lateral slip, in our report, descends downward and laterally away from the sternum deep to the central part of the breast. Because the vertical band may lead to confusion and misdiagnosis of breast tumors (in the medial part), the oblique band may lead to more confusion, as it lies deep to the central and lateral part of the breast (the commonest site for breast cancer) [19]. Unlike the medial slip, the lateral slip is expected to appear in both craniocaudal and mediolateral mammographs.

## Conclusions

We present a rare case of a sternalis muscle with multiple branches, highlighting the importance of recognizing anatomical variations in the anterior thoracic wall. A new classification system based on the branching pattern of the sternalis muscle may aid clinicians and surgeons in accurately identifying and managing such variants. Further cadaveric anatomical studies and MDCT imaging studies are warranted to better understand the prevalence and clinical significance of the sternalis muscle variations in different populations.
